# Can DNA Methylation in Peritumoral and Contralateral Breast Tissue Predict Recurrence or Second Breast Cancers?

**DOI:** 10.3390/cimb48050466

**Published:** 2026-04-30

**Authors:** Jennifer Hammer, Marie Malvaux, Louise van Drooghenbroeck, Cédric Van Marcke, Francois P. Duhoux, Martine Berliere

**Affiliations:** Breast Clinic, King Albert II Cancer Institute, Cliniques Universitaires Saint Luc, Université Catholique de Louvain, 1200 Woluwe-Saint-Lambert, Belgium; jennifer.hammer@saintluc.uclouvain.be (J.H.); marie.malvaux@cspo.be (M.M.); ldroogh2@clstjean.be (L.v.D.); cedric.vanmarcke@saintluc.uclouvain.be (C.V.M.); francois.duhoux@saintluc.uclouvain.be (F.P.D.)

**Keywords:** breast cancer, predictive factors of local recurrence, risk of contralateral breast cancer, field cancerization, tumor microenvironment, DNA methylation

## Abstract

Despite major advances in early breast cancer detection and therapeutic strategies, locoregional and distant recurrences, as well as the development of a second primary breast cancer, remain major clinical challenges. Current prognostic tools primarily rely on tumor-specific features, such as the histological grade, hormone receptor status, and proliferative index, and, more recently, on molecular signatures aimed at improving risk stratification and predicting recurrence. However, these approaches remain imperfect, and there is an urgent need to develop complementary strategies. Growing attention has been focused on the tumor microenvironment and the surrounding non-tumoral tissue, which may harbor clinically relevant molecular alterations. Emerging evidence suggests that DNA methylation changes can be detected in the adjacent and contralateral breast tissue and reflect early steps of carcinogenesis or predisposition to tumor development. This phenomenon, often referred to as field cancerization, raises new questions about the dynamics of cancer development. The aim of this work is to provide an integrative overview of DNA methylation alterations in normal breast tissue, including peritumoral and contralateral areas, and to examine their potential as predictive biomarkers of recurrence, based on the available data from tumoral tissue. In theory, these applications seem promising, but their role needs to be confirmed in large prospective trials, in order to overcome barriers to clinical implementation. The currently available evidence does not support a role for DNA methylation in the selection of locoregional and systemic treatment strategies, particularly with a view to reducing the rising number of uni- and bilateral mastectomies performed without any demonstrated survival benefit.

## 1. Introduction

Breast cancer is the most commonly diagnosed cancer among women globally and remains an important cause of cancer-related mortality [1]. Despite significant progress in early detection and therapeutic advances that have improved overall survival, recurrence remains a major clinical challenge. Recurrence comprises local recurrence, distant recurrence, and contralateral breast cancer (CBC), which is a new breast cancer. The risk of local recurrence after breast-conserving surgery (1 to 1.5% per year) and the risk of CBC (1% per year) are low among the general population of breast cancer patients [2]. However, some subgroups of patients exhibit a higher risk of local recurrence and/or CBC [3,4,5,6]. Distant recurrence occurs in up to 15 to 30% of patients, depending on the tumor subtype and initial staging [7]. Current prognostic tools primarily rely on patients’ characteristics, such as young age [8]; on tumor-specific features, such as histological grade, hormone receptor status, and proliferative index; and more recently, on molecular signatures derived from gene expression profiling [9,10,11]. Several multigene assays have been developed to improve risk stratification, including PAM50 (Prosigna), MammaPrint, and Oncotype DX [7,10,12]. While these tools have enhanced clinical decision-making, they often fall short in accurately predicting which patients will experience recurrence, particularly in early-stage or low-risk disease [13]. Current statistical risk prediction models have been used to evaluate the risk of second ipsilateral and CBC [2,14,15]. Some noteworthy models such as CBC risk and Predict CBC have been developed, based on known breast cancer risk factors, such as the breast density, the age at first birth, and the presence of a high-risk preneoplasia, with or without genetic information [14,16]. There is an ongoing debate about the improvement in clinical prediction performance using machine learning approaches compared to standard regression models for risk prediction [4,14,15]. Regardless of the methodology, improved predictive tools are essential to guide clinical decision-making. Overestimating the risk of recurrence or second breast cancer (including ipsilateral and contralateral events) may result in recommending unilateral or bilateral mastectomy for patients at a low actual risk. Conversely, underestimation can lead to inadequate surveillance or reluctance to recommend mastectomy in patients with a substantial risk. Neither is trivial. The incidence of CBC is low in the general breast cancer population, estimated at 0.25–0.4%/year for patients with ER-positive tumors treated with tamoxifen and at 0.45–0.6%/year for ER-negative tumors. However, the incidence of contralateral preventive mastectomy (CPM) is increasing, even among breast cancer patients at a low actual risk of recurrence [17]. Women diagnosed with unilateral breast cancer are increasingly opting for CPM, often due to an overestimation of their risk for CBC and the belief that CPM improves survival. However, there is no convincing evidence to support this benefit in the general population, except in cases involving high-risk mutations [16,18]. Moreover, CPM can lead to significant side effects and surgical complications. In the United States, the proportion of women with stage I-III breast cancer undergoing CPM increased from 1.8% in 1998 to 12.7% in 2012. More recent data report CPM rates reaching up to 40% in some centers. Although this increase spans all age groups, it is especially pronounced in younger women, with rates as high as 35% in this population [19,20].

In recent years, growing attention has been paid to the tumor microenvironment and the surrounding non-tumoral tissue and, more particularly, to the histologically normal peritumoral breast tissue. Emerging evidence suggests that molecular alterations, including DNA methylation changes, can be detected in these adjacent tissues and may reflect early steps of carcinogenesis or predisposition to tumor development [21,22,23,24]. This phenomenon, often referred to as “field cancerization”, challenges the traditional boundary between tumor and normal tissue and raises new questions about the dynamics of cancer development [25]. In parallel, tumor-induced remodeling of the local stroma and immune environment has been proposed as a complementary mechanism contributing to recurrence.

Among these molecular features, epigenetic modifications, particularly DNA methylation, have gained growing interest. DNA methylation is a stable and heritable mechanism that regulates gene expression and influences the chromatin structure. Aberrant methylation patterns have been detected not only in primary tumors but also in histologically benign tissue adjacent to tumors and even in the contralateral breast [21,26,27]. These alterations may indicate early field effects and hold potential as predictors of recurrence risk [27,28].

After reviewing the literature on DNA methylation in tumor samples, we examined the currently available data on DNA methylation in histologically normal tissues. A key question is whether DNA methylation alterations in histologically normal tissue can provide additional predictive value beyond tumor-based markers.

This study aims to provide an integrated overview of DNA methylation alterations in histologically normal breast tissue, including peritumoral and contralateral regions, and to evaluate their potential as predictive biomarkers for recurrence or second breast cancer. In addition to synthesizing the molecular findings, we explore the clinical implications of these alterations for prognosis, therapeutic response, and individual risk assessment and discuss the current limitations of the available evidence.

## 2. Literature Search Strategy

A narrative review of the literature was conducted using the PubMed and Scopus databases. Given the limited number of studies specifically investigating DNA methylation in histologically normal breast tissue, a PRISMA flowchart was not applied. The search strategy included the following keywords: DNA methylation in normal breast tissue, adjacent normal breast tissue, risk of recurrence, and contralateral breast cancer. Due to the limited number of relevant studies, the search period was extended from 2014 to 2025.

The references identified through this strategy were independently reviewed by three authors (J.H., M.M., and M.B.) using a two-step selection process. First, titles and abstracts were screened to exclude clearly irrelevant studies. Second, the full texts of selected articles were assessed based on predefined eligibility criteria.

Observational and interventional studies evaluating DNA methylation in normal breast tissue and its association with breast cancer risk were considered eligible for inclusion.

## 3. Field Cancerization and Tumor Microenvironment: Intertwined Concepts

### 3.1. Historical Perspective and Definitions

The concept of field cancerization was first described by Slaughter et al. in 1953, based on the observation that histologically normal epithelium adjacent to oral squamous carcinomas could contain molecular abnormalities [25]. This suggested the presence of a precancerous field extending beyond the macroscopic tumor margins. The concept has since been applied to various epithelial cancers, including breast cancer, where it refers to genetic, epigenetic, or transcriptomic alterations in histologically normal peritumoral or contralateral tissue [29].

These alterations may reflect early steps in carcinogenic processes or persistent tumor-induced changes, challenging the traditional distinction between tumor and normal tissue.

### 3.2. Field Cancerization in Breast Tissue

As described in a recent review by Gadaleta et al., several studies have identified molecular alterations, particularly in DNA methylation, in peritumoral and contralateral breast tissue, suggesting that epigenetic changes may extend beyond the primary lesion [30]. Panjarian et al. reported accelerated epigenetic aging in normal-appearing breast tissue from women with cancer compared to healthy controls [21]. Similarly, Dennis et al. observed distinct methylation patterns in tumor-adjacent, distant, and contralateral breast tissue, with intermediate alterations in clinically unaffected breasts, supporting a potential field effect [31].

In addition, pathological features such as lobular involution or mammographic density have been associated with field cancerization, suggesting that non-tumor tissue characteristics may reflect underlying carcinogenic susceptibility [30,32].

### 3.3. Pro-Recurrence Tumor Microenvironment

Local recurrence may also be influenced by a pro-recurrence tumor microenvironment (TME), composed of stromal, immune, and extracellular components that support tumor progression and may persist after resection [33,34,35]. This complex ecosystem involves dynamic interactions between these components that contribute to tumor progression and recurrence. Stromal alterations, such as stromal disruption, have been associated with increased breast cancer risk [36,37]. Li et al. further emphasized the importance of these interactions in shaping tumor behavior and their potential as targets for future therapeutic strategies, particularly through the modulation of NK cell activity [38].

### 3.4. Complementary Theories or Overlapping Realities

While conceptually distinct, field cancerization and the pro-recurrence tumor microenvironment are increasingly viewed as complementary biological processes rather than mutually exclusive explanations of cancer development and relapse. Studies have demonstrated DNA methylation alterations in distant benign breast tissue, supporting a field-based susceptibility model [31,35], while stromal and immune changes further suggest a role for tumor-induced microenvironmental remodeling [33,34,35].

**Figure 1:**
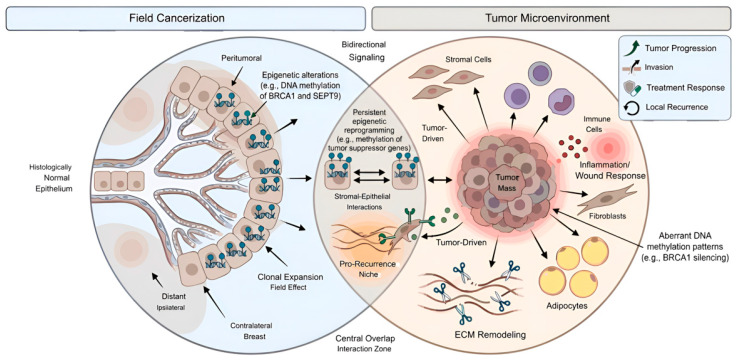
Schematic representation of field cancerization and tumor microenvironment interactions in breast tissue. Field cancerization primarily involves epigenetic alterations occurring in histologically normal breast epithelium, which may extend beyond the peritumoral region to distant ipsilateral or contralateral tissue, reflecting a spatially distributed susceptible field. These alterations include DNA methylation changes affecting genes such as BRCA1 and SEPT9. In contrast, the tumor microenvironment is shaped by tumor-driven remodeling of stromal, immune, and extracellular components, which may persist after tumor resection and contribute to disease progression. The central overlap highlights dynamic stromal–epithelial interactions and persistent epigenetic reprograming, including methylation of tumor suppressor genes, which together may create a pro-recurrence niche. This framework illustrates the interplay between tissue susceptibility, tumor evolution, and relapse. Created in BioRender. Berliere, M. (2026). BioRender.com/cwivxp2.

The co-occurrence of epithelial and stromal alterations in premalignant breast tissue supports the existence of interconnected pathways in early breast cancer development [37]. Together, these findings suggest that tumorigenesis and recurrence arise from the interplay between pre-existing field effects and microenvironmental changes [39,40]. These concepts are summarized in Figure 1.

## 4. DNA Methylation in Tumor Samples: Brief Summary

Although the primary focus of this review is on DNA methylation alterations in histologically normal peritumoral and contralateral breast tissue, tumor-based methylation studies are included as an essential reference framework. Tumor tissue represents the most extensively characterized context, with more robust clinical annotation and stronger associations with recurrence and prognosis. Therefore, summarizing tumor-associated DNA methylation patterns provides a necessary benchmark to contextualize the emerging and largely exploratory findings in normal breast tissue, while acknowledging that direct extrapolation from tumor to normal tissue remains limited and requires cautious interpretation.

### 4.1. Mechanisms and Early Alterations

DNA methylation, the covalent addition of a methyl group to the 5′ position of cytosine in CpG dinucleotides, is a fundamental epigenetic mechanism involved in gene regulation, chromatin structure, and genomic stability. In healthy tissue, DNA methylation contributes to tissue-specific gene expression and cellular differentiation. In breast cancer, aberrant methylation patterns have been extensively documented, including promoter hypermethylation of tumor suppressor genes (e.g., CDH1, RASSF1A, BRCA1) and global hypomethylation, which may activate oncogenes or transposable elements and promote genomic instability through interference with the protective function of telomeres and centromeres; this instability leads to increased tumor heterogeneity and thereby fuels cancer evolution [41,42,43,44].

These methylation abnormalities are subtype-specific: triple-negative breast cancers (TNBCs) exhibit distinct methylation landscapes compared to luminal or HER2-positive subtypes [25]. A genome-wide methylation profiling study has shown that DNA methylation alterations occur early in tumorigenesis, even before histological transformation, and may reflect an altered tissue environment or pre-existing susceptibility to malignancy [44]. The stability and reversibility of methylation markers make them attractive candidates for biomarker development and therapeutic targeting [42,45].

### 4.2. Subtype-Specific Methylation Signatures and Their Clinical Utility

Beyond global methylation dysregulation, distinct DNA methylation profiles have been associated with breast cancer subtypes and clinical behavior. High-throughput studies have revealed that triple-negative breast cancers (TNBCs) typically exhibit global hypomethylation with focal promoter hypermethylation, while luminal tumors show more uniform hypermethylation across defined loci [44,46]. These findings highlight the epigenetic heterogeneity of breast cancer and support the use of methylation-based classification systems complementary to gene expression-based subtyping [46,47,48,49].

Large-scale analyses have reinforced the prognostic potential of DNA methylation by identifying multi-CpG signatures capable of stratifying breast cancers into biologically and clinically distinct groups. Zhang et al., for example, analyzed over 5000 CpG sites across clinical tumor specimens and defined methylation-driven clusters differing in proliferation, immune evasion, and metastatic potential [46]. Such epigenetic stratification holds particular value for TNBCs, where conventional molecular subtyping remains limited by high intragroup heterogeneity.

These findings suggest that integrating methylation profiles into existing classification systems could enhance the precision of prognostic assessment and therapeutic decision-making.

### 4.3. Implications for Treatment Response

DNA methylation can also impact treatment outcomes. Hypermethylation-induced silencing of genes involved in DNA repair (e.g., BRCA1), oxidative stress regulation (SOD3), and growth control (BMP6, BMP2) has been linked to resistance to chemotherapy and endocrine therapy, particularly in aggressive subtypes such as TNBC [42,46]. These findings support the potential use of methylation profiling to identify patients less likely to respond to specific treatments. An interesting example is the particular case of BRCA1. Promoter hypermethylation of BRCA1 has been extensively studied [39,50,51]. The inactivation of BRCA1 by epigenetic alterations is a critical event in breast tumorigenesis, which may potentially be used as a prognostic marker for patients with breast cancer. Wu et al. systematically reviewed the promoter methylation of BRCA1 and its relationship to the clinical outcomes of breast cancer patients. In a meta-analysis of nine studies encompassing 3205 patients, BRCA1 methylation was found to be significantly correlated with a poor overall survival of breast cancer, with a combined HR (95% CI) of 2.02 (1.35–3.03). After adjusting for potential confounders using the Cox regression model, the pooled HR (95% CI) of BRCA1 methylation on patients’ overall survival was 1.38 (1.04–1.84). When disease-free survival was used as the outcome, the combined HR (95% CI) was 2.89 (1.73–4.83) in a univariate analysis and 3.92 (95% CI 1.49–10.32) in a multivariate analysis, respectively. Subgroup analyses of specimen types revealed that the pooled HR (95% CI) for overall survival was 1.48 (1.22–1.81) when using formalin-fixed paraffin-embedded (FFPE) specimens and 1.38 (0.16–11.84) when using fresh-frozen tissues. As for disease-free survival, the pooled HR (95% CI) was 2.47 (1.33–4.58) when using FFPE specimens and 2.78 (1.47–5.28) when using fresh-frozen tissues. This meta-analysis provides evidence that BRCA1 methylation is a negative prognostic factor in breast cancer.

### 4.4. Tumor Tissue vs. Histologically Normal Tissue: Current Level of Evidence

Evidence linking DNA methylation patterns to prognosis and recurrence is currently strongest in tumor tissue, where cohorts are larger, follow-up is more frequently available, and signatures have been more extensively evaluated. In contrast, methylation alterations reported in histologically normal peritumoral or contralateral tissue remain largely exploratory and should be considered hypothesis-generating, as most studies are retrospective, include limited sample sizes, and are affected by confounding factors such as cell-type heterogeneity and the sampling distance. Accordingly, while tumor-based methylation signatures provide an important benchmark, the translation of normal-tissue methylation patterns into clinical decision-making is not yet supported and will require prospective validation.

To clarify the differences in evidence level and translational maturity, a comparative overview of tumor-based and normal-tissue-based DNA methylation biomarkers is provided in Table 1.

## 5. Discussion: Clinical Implications and Translational Perspectives

### Prognostic and Predictive Value

The traditional histopathology-driven classification of breast cancers has been integrated with molecular markers to better evaluate the features of each type of breast cancer. The need for genomic tests to estimate the exact risk of recurrence and prognosis of the individual patients led to the development of first- and second-generation prognostic gene signatures. Mammaprint, Prosigna (PAM 50 based) and Oncotype DX gene expression assays have been used in many clinical trials and are now used in clinical practice in an attempt to guide treatment decisions [52,53]. However, as they are based on the mRNA abundance of several selected genes, these gene expression profiles only capture specific parts of the state of the tumor at a given timepoint.

DNA methylation is potentially more stable, as it is independent of current transcriptional activity. As such, epigenomic research has the potential to contribute to breast cancer subtyping and survival prediction, even though there is currently no consensus as to whether DNA methylation may be used as an additional tool to subtype breast cancer and/or predict prognosis. Different studies have been elaborated in different contexts including methylation-based subtypes, research for candidate genes, and whole-genome screening [54,55,56,57,58,59,60,61]. In general, these studies are retrospective, small, and lack external validation. The clinical utility of many proposed methylation signatures remains uncertain, and larger studies with external validation are warranted [61]. The main recent studies published in the literature with data on survival are presented in Table 1. Of note, most published studies that have investigated the link between tumor DNA methylation and breast cancer survival suffer from a lack of replication. Table 2 presents studies evaluating the association between DNA methylation in breast tumors and survival outcomes.

However, many of these studies have severe limitations and unclear aspects. Most used public databases (TCGA) to identify DNA methylation involved in breast cancer development. Information about methylation was captured by the Illumina Epic Methylation Array, not a genome-wide analysis. In some studies, no external validation was performed [56,59].

Different studies have investigated the association between tumor DNA methylation and breast cancer survival. They are divided into methylation-based subtypes, candidate genes, and genome-wide studies. 

For studies based on different subtypes, most analyses were based on a small number of deaths, limiting the reliability of these findings. The same observations were present in studies with DNA methylation in candidate genes associated with breast cancer survival and genome-wide studies of DNA methylation markers. Moreover, adjustment for clinical prognostic factors was inconsistently considered, while the prognostic power can be strongly improved by combining this with information on molecular data. The lack of standardization limits the robustness and thus the clinical applicability of the findings. The studies considered discrimination metrics such as ROC-AUC or the C index, which may overstate the model performance without assessing how well the predicted risks align with observed outcomes.

Another point is that research studies have compared breast cancer samples with “normal adjacent” tissue, but several reports have shown that adjacent normal tissue is not normal tissue.

## 6. DNA Methylation in Histologically Normal Breast Tissue

### 6.1. DNA Methylation in Normal Breast Tissue

The vast majority of studies on DNA methylation in normal breast tissue rely on this tissue as a non-cancerous reference in tumor-focused studies investigating epigenetic alterations in breast cancer. The remarkable study performed by Dennis et al. illustrates this concept [31]. Their results suggest that benign tissues from breast cancer patients differ substantially from healthy donor breast tissue. Definitively, normal breast tissue, whether adjacent or contralateral, is not normal in the case of breast cancer.

Crucially, the epigenetic changes are not confined to the tumor itself. Studies have demonstrated their presence in adjacent or even contralateral tissue, supporting the feasibility of sampling strategies—to assess the recurrence risk and personalize follow-up [30,63]. While still exploratory, these approaches open promising avenues for individualized monitoring and molecular risk stratification.

Different studies [64,65,66] (Johnson, Muse, Teschendorff) in women without breast cancer have demonstrated that DNA methylation profiles in solid breast tissue are associated with age, a well-known breast cancer risk factor. This finding was confirmed by Muse et al. [65], who observed that DNA methylation changes with age occur in regulatory regions, suggesting a loss of cellular state control as an individual ages. Furthermore, they demonstrated additional support for a link between age-related DNA methylation and cancer, as age-related CpG sites were more likely to exhibit more alterations in both pre-invasive and invasive breast cancer. Together, their findings suggest that DNA methylation changes in aging shift the epigenetic state towards a compromised molecular phenotype, creating a novel link between the risk factor of age and the potential origins of disease in breast cancer.

The heterogeneity and inconsistency in the definition of histologically normal tissue across studies is an important point that must be emphasized. In many studies, the distance between the primary tumor and the tissue sample that was collected and classified as “normal” is not mentioned, although this may influence the epigenetic alterations observed, as well as inter-study comparison and the interpretation of the results. If the breast samples analyzed are not taken at a wide margin—it seems that at least 3 cm away from the invasive lesion is necessary—contamination of adjacent normal samples by neighboring cancer cells may still occur and may influence the observed epigenetic alterations and the DNA methylation profiles.

### 6.2. Potential for Non-Invasive or Minimally Invasive Monitoring

To translate these results into clinical practice, it would be desirable to be able to estimate the risk of developing breast cancer using less invasive methods. The next step is to find factors in blood, saliva, or urine that accurately reflect the methylation changes in breast tissue associated with the development of breast cancer, in order to potentially develop risk models that can be easily used by the largest number of women globally.

Some studies have shown disappointing results: Visvanathan et al. examined whether DNA methylation assessed in random fine needle aspirates could be used to inform breast cancer risk [67]. In 20 women with invasive breast cancer scheduled for surgery at the Johns Hopkins Hospital, the cumulative methylation status was assessed by random fine needle aspiration (rFNA), performed on the tumor, tumor-adjacent normal tissues, and all remaining quadrants. Unfortunately, they concluded that the diagnostic accuracy of methylation based on rFNA alone to detect premalignant lesions or at-risk quadrants is poor and should not be used to evaluate cancer risk.

Blood represents another source that allows for minimally invasive monitoring.

The role of gene-specific methylation in peripheral blood as a marker of breast cancer risk is under evaluation, because peripheral blood is a comparatively more accessible biospecimen. Only a few studies have been conducted to perform this evaluation, and further investigations are required to determine whether DNA methylation may be a new tool to predict the risk of breast cancer, because challenges include inconsistent findings across studies [39,68,69].

### 6.3. Alterations in DNA Methylation Identified in Histologically Normal Breast Tissue

Alterations in DNA methylation identified in histologically normal breast tissue—particularly in peritumoral or contralateral regions—are increasingly recognized as clinically relevant. Nevertheless, further studies utilizing specimens from biobanks are required to determine the extent to which molecular alterations identified in peritumoral or contralateral tissues contribute to future tumorigenesis [21,31]. Studying DNA methylation alterations seems to be fertile ground for revolutionizing the global therapeutic and preventive approach to breast cancer. Importantly, a substantial proportion of breast cancer patients undergo oncoplastic procedures and contralateral breast symmetrization during conservative surgery, which presents a unique opportunity to prospectively assess DNA methylation changes not only in the peritumoral parenchyma but also in more distant regions of the same breast and in the contralateral breast. These future studies will need to integrate clinical information such as environmental exposures, disease evolution, pathological characteristics, and molecular alterations within the cancerized field. Several DNA methylation alterations identified in histologically normal breast tissue—particularly in peritumoral and contralateral regions—have been associated with tumorigenesis and disease progression. These include methylation changes affecting genes such as NEFM, SOD3, SEPT9, BMP6, and BRCA1, which have been linked to recurrence, metastasis, or poor prognosis in tumor-based studies [31,48,49,62,70]. Although these markers are well characterized in tumor tissues, their presence in histologically normal tissue suggests a potential role as early indicators of cancer susceptibility or recurrence risk. However, most studies investigating DNA methylation in histologically normal breast tissue lack external validation, highlighting a major limitation for clinical translation. Table 3 summarizes key methylation alterations identified in histologically normal breast tissue, along with their validation status.

The studies using normal breast samples, adjacent to breast tumors, highlight the fact that histologically normal breast tissue is not “totally normal”, because it contains epigenetic abnormalities and could, therefore, be used as a marker of recurrence.

### 6.4. Toward Risk Stratification and Primary Prevention

Recent findings also open the door to early risk assessment, even before cancer develops. Mapping genetic and epigenetic changes in normal breast tissue at risk of neoplastic transformation is critically important for understanding oncogenesis and identifying causal drivers for cancer risk prediction and recurrence.

Studies have shown that histologically normal tissue from women with breast cancer exhibits signs of accelerated epigenetic aging or promoter hypermethylation of tumor suppressor genes compared to tissue from healthy donors [21,31,71,74]. These patterns suggest that the normal epithelium may reflect an underlying predisposition to malignancy or the current effects of carcinogenic factors.

However, most of these studies are not prospective, do not integrate molecular data with clinical follow-up, and therefore, do not directly assess the risk of recurrence or the risk of contralateral breast cancer [71,74].

**Figure 2:**
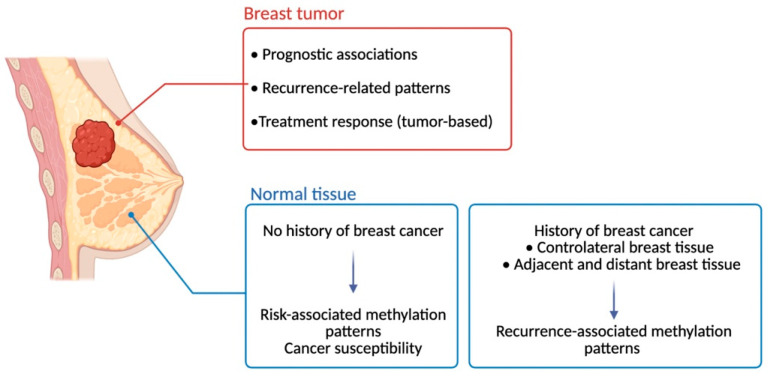
DNA methylation in breast tissue: current and emerging clinical contexts. DNA methylation profiling in breast tumors has been associated with prognostic information and treatment response in retrospective cohorts. In contrast, methylation alterations identified in histologically normal breast tissue, including peritumoral and contralateral samples, remain largely exploratory and hypothesis-generating. While these patterns may reflect cancer susceptibility or field cancerization effects, their clinical utility has not yet been established and requires validation in large prospective studies. Created in BioRender. Berliere, M. (2026). BioRender.com/wv6xsqy.

While not yet applied in clinical practice, integrating such epigenetic markers into existing risk models could allow for individualized prevention strategies [73]. Tissue sampling from the contralateral breast or high-risk cohorts (e.g., BRCA1/2 mutation carriers) may help stratify patients according to their molecular susceptibility, potentially guiding screening intervals or preventive interventions. However, further research is needed to validate these approaches and assess their feasibility in prospective settings [30,75]. Furthermore, future in vitro and in vivo studies could also assess whether specific demonstrated or suspected carcinogenic factors are associated with the accumulation of distinct methylation changes, as already demonstrated with genome-wide mutation patterns [76,77,78]. Ongoing and future clinical applications of DNA methylation are illustrated in Figure 2.

In the general breast cancer population, predicting the CBC risk remains challenging due to the moderate performance of the currently known risk factors. Including additional risk factors is necessary to improve the performance of the existing predictive models. In the context of large-scale prospective studies, adding a methylation score to predictive models represents an interesting option to explore.

Nevertheless, the risk of low specificity leading to overtreatment must be addressed, especially with the currently insufficient available data. This risk seems particularly important among low-risk non-mutated breast cancer patients whose tumors express hormone receptors. Among these patients, the benefits of prolonged endocrine therapy and the addition of CDK4/6 inhibitors on locoregional recurrence and contralateral breast cancer risk should be carefully evaluated within a risk–benefit framework to determine the potential value of a more aggressive surgical strategy.

## 7. Limitations, Challenges, Perspectives, and Conclusions

### 7.1. Limitations and Challenges to Clinical Implementation

Most of the available studies are based on relatively small datasets, and several analytical aspects vary across studies, such as the variability in tumor purity. In addition, correlations with clinical data, especially long-term follow-up, remain limited, and the methodological heterogeneity highlights the need for standardization to allow reliable comparisons. Furthermore, some studies lack external validation.

In normal breast tissue, the low signal-to-noise ratio of DNA methylation changes may introduce some substantial technical artifacts such as batch effects, which can reduce the accuracy of clustering and data interpretation.

### 7.2. Perspectives

Aberrant DNA methylation is recognized as involved in the early steps of cancer development, progression, and recurrence. Various methylation markers have been investigated, with limited success to date. The concept of a single methylation marker applicable across multiple cancer types has therefore been proposed, referred to as the Universal Cancer Only Marker (UCOM) [79].

Recently, Dong et al. reported preliminary findings on SIX6, a homeobox transcription factor, which was found to function as a tumorigenesis regulator [79]. They demonstrated that SIX6 is consistently hypermethylated in cancer samples compared with normal tissues. In addition, SIX6 may serve as a biomarker for tracing metastasis, as its methylation levels can be detected in positive lymph nodes, including in breast cancer. Furthermore, the methylation level of SIX6 may help guide surgical resection, as it appears to decrease with increasing distance from the tumor lesion.

### 7.3. Conclusions

Although tools exist to estimate the risk of local recurrence following breast-conserving surgery, as well as the risk of contralateral breast cancer and distant metastases, their predictive value remains imperfect [3,9,11,34,80]. The development of novel strategies is therefore essential. In this context, epigenetic markers, particularly DNA methylation, could theoretically represent a promising avenue to improve risk assessment.

The biobanking of human material from mastectomies and contralateral breast symmetrization procedures allows the prospective evaluation of DNA methylation alterations in the peritumoral and distant regions of the same breast, as well as in the contralateral breast. Decoding the epigenetic landscape of histologically normal breast tissue may represent a promising approach to refine recurrence risk stratification and support clinical decision-making. However, the preliminary findings require validation in large-scale prospective studies or registries with robust statistical frameworks to confirm their clinical utility.

The currently available evidence does not support a role for DNA methylation in guiding the selection of locoregional and systemic treatment strategies, particularly with the aim of reducing the increasing number of uni- and bilateral mastectomies performed without any demonstrated survival benefit.

## Figures and Tables

**Table 1:** Comparison of evidence level, study design, limitations, and clinical readiness between tumor-derived and normal-tissue-based DNA methylation biomarkers.

Normal Peritumoral/Contralateral Tissue Biomarkers	Tumor Tissue-Based Biomarkers	Aspect
Histologically normal epithelium and/or stroma	Malignant epithelial tissue	Biological context
Low–emerging (small, exploratory studies)	Moderate to high (multiple retrospective cohorts; some validation)	Evidence level
Retrospective, limited cohorts; spatial heterogeneity	Retrospective, tumor-focused profiling (e.g., TCGA)	Typical study design
Limited follow-up and clinical endpoints	Recurrence, survival, treatment response frequently available	Clinical annotation
Peritumoral/contralateral methylation patterns [31,37]	BRCA1, SEPT9, NEFM, SOD3	Representative examples
Cell-type heterogeneity; lack of prospective validation	Platform variability; limited prospective validation	Main limitations
Not yet clinically validated	Research use; limited translational maturity	Clinical readiness

Abbreviations: TCGA, The Cancer Genome Atlas; BRCA1, Breast Cancer Gene 1; SEPT9, Septin 9 Gene; NEFM, Neurofilament Medium Chain; SOD3, Superoxide Dismutase 3 Gene.

**Table 2:** Studies evaluating the association between DNA methylation in breast tumors and survival outcomes.

Statistical Analysis/Key Results	Validation	CpGs in Signature	Outcomes	Cohort	Authors
Whole-genome methylation; 268 tumors, 4 normal samples	External validation (ROC AUC = 0.64)	8	OS	TCGA	Hao et al., 2017 [62]
Cox regression, 1076 tumors	None	NR	RFS (5 years)	GDC	Ren et al., 2018 [54]
Regression model; 786 tumors, 97 normal samples	Eternal validation (ROC AUC = 0.94; HR = 2.08)	16	OS	TCGA	
Cox regression; 1336 samples (148 normal)	None	7	OS	TCGA	Tao et al., 2020 [56]
Cox regression; 1438 tumors	External validation (ROC AUC 0.62–0.65)	28	OS (1–10 years)	TCGA	Liu et al., 2020 [57]
Array + alternative assay (r = 0.495; *p* = 0.0009)	Technical validation	Panel	RFS	IBCSG VIII–IX (TNBC)	Fackler et al., 2020 [58]
Pre-/post-NAC biopsies	None	4	OS	Norwegian cohort	Pedersen et al., 2022 [59]
Cox regression; 158 TNBC samples	External validation (ROC AUC = 0.64)		OS	TCGA (TNBC)	Peng et al., 2020 [60]
Cox regression; 425 tumors	External validation (ROC AUC = 0.63)	2–3 signatures	Breast cancer-specific mortality	MCCS	Zarean et al., 2025 [61]

Summary of cohort characteristics, outcomes, statistical analyses, and the number of CpG sites included in prognostic methylation signatures. Abbreviations: TCGA, The Cancer Genome Atlas; GDC, Genomic Data Commons; MCCS, Melbourne Collaborative Cohort Study; OS, Overall Survival; RFS, Recurrence-Free Survival; TNBC, Triple-Negative Breast Cancer; ROC AUC, Receiver Operating Characteristic Area under the Curve (NR = not reported).

**Table 3:** Key DNA methylation alterations identified in histologically normal, peritumoral, or contralateral breast tissue.

Validation	Methylation Marker/Alteration	Technique	Cohort	Authors
External validation	EpiTOC (age-related methylation)	Illumina 450 K	Cancer-free women + adjacent	Johnson et al., 2017 [64]
External validation	Differential methylation	Illumina 450 K	Normal + adjacent	Gao et al., 2018 [71]
No external validation	110 genes	Illumina 27 K	TCGA	Avraham et al., 2014 [72]
External validation	Identification of two genes *FAM8317* and *NEK2* Differential methylation	MethylCap + RNA-seq	Healthy women	Marino et al., 2022 [73]
External validation	Identification of 772 hypermethylated CpGs Cell-type-independent alterations	Illumina 450 K	Normal + breast milk	
External validation	Stepwise methylation	Illumina EPIC	Tumor, peritumoral, contralateral	Dennis et al., 2025 [31]

Summary of key DNA methylation alterations identified in histologically normal, peritumoral, or contralateral breast tissue, along with their validation status and study characteristics. Abbreviations: FDR, false discovery rate; EpiTOC, epigenetic timing of cancer.

## Data Availability

No new data were created or analyzed in this study. Data sharing is not applicable to this article.

## Abbreviations

The following abbreviations are used in this manuscript:

CBC	Contralateral Breast Cancer
CPM	Contralateral Preventive Mastectomy
ER	Estrogen Receptor
BRCA1 and BRCA2	Breast Cancer Gene 1 and Breast Cancer Gene 2
CDH1 or CADH1	Cadherin 1 Gene
RASSFIA	Ras Association Domain Family Member 1
TNBC	Triple Negative Breast Cancer
NEFM	Neurofilament Medium Chain
SOD3	Superoxide Dismutase 3 Gene
SEPT9	Septin 9 Gene
BMP6	Bone Morphogenetic Protein 6
BMP2	Bone Morphogenetic Protein 2
LRR	Local Recurrence Risk
CBCR	Contralateral Breast Cancer Risk
TCGA	The Cancer Genome Atlas
MCCS	The Melbourne Collaborative Cohort Study
GDC	Genomic Data Commons
DRR	Distant Recurrence Risk
OS	Overall Survival
RFS	Recurrence-Free Survival
rFNA	Random Fine Needle Aspiration
FDR	False Discovery Rate
ROC AUC	Receiver Operating Characteristic Area Under the Curve
EPITOC	Epigenetic Timing of Cancer
